# “When I Sleep Poorly, It Impacts Everything”: An Exploratory Qualitative Investigation of Stress and Sleep in Junior Endurance Athletes

**DOI:** 10.3389/fpsyg.2021.618379

**Published:** 2021-02-15

**Authors:** Maria Hrozanova, Kristian Firing, Frode Moen

**Affiliations:** ^1^Faculty of Medicine and Health Sciences, Department of Neuromedicine and Movement Science, Norwegian University of Science and Technology, Trondheim, Norway; ^2^Faculty of Social and Educational Sciences, Department of Teacher Education, Norwegian University of Science and Technology, Trondheim, Norway; ^3^Faculty of Social and Educational Sciences, Department of Education and Lifelong Learning, Norwegian University of Science and Technology, Trondheim, Norway

**Keywords:** mental stress, psychological load, recovery, semi-structured interview, restitution, cross-country skiing, biathlon

## Abstract

On their journeys toward senior athletic status, junior endurance athletes are faced with a multitude of stressors. How athletes react to stressors plays a vital part in effective adaptation to the demanding, ever-changing athletic environment. Sleep, the most valued recovery strategy available to athletes, has the potential to influence and balance athletic stress, and enable optimal functioning. However, sleep is sensitive to disturbances by stress, which is described by the concept of sleep reactivity. Among athletes, poor sleep quality is frequently reported, but our understanding of the associations between stress and sleep in junior athletes is currently incomplete. The present study therefore investigated the themes of stress and sleep, and the associations between these variables with the use of in-depth semi-structured interviews in six junior endurance athletes (three men and three women, mean age 17.7 ± 0.5 years). Data was analyzed qualitatively based on the Grounded Theory. The qualitative material was supplemented with quantitative data on subjective sleep quality (Pittsburg Sleep Quality Index), sleep reactivity (Ford Insomnia Response to Stress Test), and mental strain (visual analog scale). The main results showed that stress could be differentiated into relevant stressors (encompassing poor performance, uncertainty in relation to training, school, daily hassles, and sleep) and reactions to stress (with sub-categories facilitative and maladaptive). Sleep could be differentiated into sleep benefits (encompassing energy levels and athletic functioning) and sleep quality (with sub-categories satisfactory and inadequate). All athletes identified relevant stressors, and all athletes were aware of the benefits of sleep for athletic functioning. However, athletes formed two distinctive categories based on the interactions between stress and sleep: three exhibited facilitative reactions to stress and good sleep quality, as well as low sleep reactivity, and low mental strain. The remaining participants exhibited maladaptive reactions to stress and poor sleep quality, as well as high sleep reactivity and high mental strain. Conceptualizing sleep quality based on the evaluation of stressors, reactions to stress, degree of mental strain, and the propensity to stress-related sleep disturbance may offer a plausible explanation for why the occurrence of stressors leads to poor sleep quality in some athletes, but not others.

## Introduction

Junior athletes who possess ambitions of an elite sports career face obstacles and challenges on their way toward goal attainment. This journey may offer a deep sense of purpose, belonging and motivation; but there is also an ever-present risk of disappointments, setbacks, and adversity (Houltberg et al., 2018; Reardon et al., 2019). The ability to manage the stressful aspects of athletic careers, and the ability to adequately recover on a continuous basis (Kellmann, 2010), i.e., the concepts of stress and sleep, are central requirements for optimal athletic functioning. Research in athletic populations has previously shown that affective and cognitive aspects of stress lead to disrupted sleep (Lastella et al., 2014; Juliff et al., 2015; Hrozanova et al., 2020). However, a thorough understanding of the associations between the stress and sleep is lacking.

### Stress in the Athletic Setting

Stress is defined as a dynamic state in which the homeostasis of an organism is perceived to be threatened by relevant stressors (Chrousos and Gold, 1992). In the athletic setting, relevant stressors belong to three categories: the *double burden*, or stress due to combining sport with duties such as education; the *sport-specific demands*, or psycho-physiological stress related to sport participation; and *conditions*, or stress from unfavorable structures within the team or organization (Nixdorf et al., 2015). Importantly, the occurrence of a relevant stressor marks the activation of the stress response, which aims to restore the homeostatic state of the organism (Chrousos and Gold, 1992; Schneiderman et al., 2005). The stress response is a dynamic process, and its outcomes are unique **in** each individual (Ellis et al., 2006). To illustrate, when meeting an identical stressor (e.g., an important competition), one athlete may experience little change from baseline, keeping a cool composure, while another may experience high levels of nervousness and heart palpitations. According to the Biopsychosocial model of challenge and threat (BPS, Blascovich, 2008), the outcome of the stress response is dependent on a rapid evaluation of immediate situational demands and available coping resources. This evaluation process is triggered immediately after the activation of the stress response (Blascovich and Tomaka, 1996). Individuals who perceive the situation to be within own personal capabilities exhibit a facilitative stress response, i.e., *challenge*. Those who perceive the situation as exceeding their personal resources exhibit a maladaptive stress response, i.e., *threat* (Tomaka et al., 1993; Blascovich et al., 2001). In this way, the evaluation of the situational demands and available coping resources determines the outcome of the stress response.

Facilitative and maladaptive stress responses are associated with different psychological processes and reactions to stress. Facilitative stress responses typically lead to positive emotional states, such as challenge, energy and excitement (Tomaka et al., 1993), an active approach to positive goals and overall better performance (Fletcher and Sarkar, 2012). Such facilitative reactions are typical for athletes with high *mental resilience*—a safeguarding attribute against the development of psychopathology in times of adversity (Hjemdal et al., 2006; Fletcher and Sarkar, 2012). In contrast, maladaptive stress responses lead to negative emotional states, and avoidant, unfocused behaviors (Blascovich, 2008). An important role is played by psychological processes such as worry and rumination, collectively termed *preservative cognition*. Preservative cognition is “the repeated or chronic activation of the cognitive representation of stress-related content” (Brosschot et al., 2005, p. 1,045). Preservative cognition represents a common maladaptive reaction to stress, and has the potential to interrupt the resolution of the stress response, leading to its prolonged activation (Brosschot et al., 2005).

### Sleep Quality in Athletes, and Associations to Stress

Sleep is a crucial component of athletic recovery (Nedelec et al., 2015; Kellmann et al., 2018). Athletic recovery is enabled by multiple sleep-dependent processes, spanning the cognitive, emotional, musculoskeletal, behavioral, immune, metabolic, endocrine and glymphatic systems (for a review, see Dresler et al., 2014; Krueger et al., 2016). However, if sleep is to facilitate athletic recovery, *sleep quality* (i.e., perceived quality of obtained sleep and daytime functioning, defined by Harvey et al., 2008) has to be adequate over time (for a review, see Venter, 2012). Sleep quality may be measured with the use of standardized questionnaires, such as the Pittsburgh Sleep Quality Index (PSQI, Buysse et al., 1989). Despite the importance of sleep for athletic functioning (Kellmann et al., 2018), athletes' reports of poor subjective sleep quality (PSQI > 5, Buysse et al., 1989), during the competitive as well as preparatory phases of the season, vary from ~28% (Tuomilehto et al., 2017; Hoshikawa et al., 2018; Hrozanova et al., 2019) to ~55% (Samuels, 2008; Fietze et al., 2009; Swinbourne et al., 2016).

Athletes' poor sleep quality may be anchored in the many facets of the stress response, including the evaluation and reaction to stressors. In this context, the key mechanism may involve *sleep reactivity* (Drake et al., 2014). Sleep reactivity refers to the propensity to exhibit sleep disturbance in response to stress (Drake et al., 2014). Sleep reactivity has been associated with two sleep-disrupting phenomena: preservative cognitions and hyperarousal. Preservative cognitions likely play a crucial, mediating role in the association between stress and sleep, both in insomniacs (Baglioni et al., 2011) and healthy sleepers (Fernandez-Mendoza et al., 2010; Drake et al., 2014). The persistent worries and ruminations characteristic of preservative cognitions may lead to hyperarousal: The overactivation of neurobiological and psychological systems, which may interfere with sleep initiation and maintenance (Kalmbach et al., 2018b). This is often seen in individuals with high sleep reactivity (Kalmbach et al., 2018b). When preservative cognitions accompany high sleep reactivity, sleep disturbances such as insomnia may occur (Drake et al., 2014).

A variety of studies have implicated the subjective evaluation of stressors, hyperarousal and preservative cognitions in sleep disruptions or poor sleep quality (Morin et al., 2003; Thomsen et al., 2003; Baglioni et al., 2010; Drake et al., 2014; Palagini et al., 2018). However, investigations of sleep reactivity in athletes received little attention. In one study, elite athletes were significantly less sleep reactive than sub-elite and non-athletic controls (Gupta et al., 2017). The authors argued that the low degree of sleep reactivity among elite athletes may be because “… the impact of these challenges [sleep dysfunction] is offset by constitutional resilience and/or the ability to use compensatory strategies….” As junior (12–18 years old) athletes possess significantly worse ability to cope with adversity than senior athletes (aged 25 years and older) (Bebetsos and Antoniou, 2003), the investigation of the associations between sleep and stress seems especially relevant among junior athletes. Indeed, mental resilience may not only be important in stress responses, but also in the associations between stress and sleep. Compared to good sleepers, mental resilience was found to be lower in subjects with insomnia, who also exhibited elevated sleep reactivity and hyperarousal (Palagini et al., 2018). In athletes, a large cross-sectional study found that mental resilience predicted good sleep quality, while perceived stress and preservative cognitions (e.g., worry) predicted poor sleep quality (Hrozanova et al., 2019). Furthermore, sleep onset latency was found to increase and sleep quality to deteriorate during competitions (Lastella et al., 2014; Juliff et al., 2015), periods associated with higher stress-related somatic and cognitive arousal (Filaire et al., 2001).

The relationships between stress and sleep may be bi-directional (Kahn et al., 2013; Kalmbach et al., 2018a), suggesting that the exposure to stress may contribute to the development of a reciprocal, negative cycle between stress and sleep. In their review on the impact of stress on sleep, Kalmbach et al. (2018a) proposed that in individuals with high sleep reactivity, preservative cognitions may trigger arousal incompatible with sleep, and that the inability to sleep when stressed may offer further opportunity to engage in preservative cognitions, escalating and deepening the experience of sleep disturbance. Research has begun to provide evidence of such bidirectional relationships also in athletes. In athletes who were poor sleepers, increases in sleep onset latency were associated with higher subsequent mental strain (Hrozanova et al., 2020). Increased sleep onset latencies may have been caused by preservative cognitions at bedtime (Wuyts et al., 2012), which may have in turn contributed to increases in mental strain (Galambos et al., 2009), possibly leading to further sleep disturbance (Kalmbach et al., 2018a). However, further research is needed in order to elucidate the bidirectionality between stress and sleep.

### The Current Study

The interactions between stress and sleep are complex and dynamic, and highly relevant to the functioning of junior athletes. The individual stress response plays a vital part in effective adaptation to the demanding and permanently changing athletic environment. Sleep fulfills important recovery-enabling functions (e.g., optimization of physiological, psychological, musculoskeletal, immune, metabolic, endocrine and glymphatic systems), but quantitative investigations have shown that athletes may have trouble getting sleep of adequate quality. This is striking, since important links between sleep and improved athletic performance have been identified (reviewed in Walsh et al., 2020). The aim of the current study was to qualitatively explore athletes' understanding of stress and sleep and to identify and explain associations between these variables in a sample of junior endurance athletes, taking into consideration athletes' quantitative accounts of sleep quality, sleep reactivity, as well as cognitive and affective mental strain.

## Methods

### Design and Procedure

There are marked gaps in the current understanding of the interactions between sleep and stress in junior athletes. Existing research points to the importance of the evaluation of the stressful situation in terms of situational demands and available coping resources (Blascovich, 2008), and research from non-athletic and insomniac populations suggests the relevance of sleep reactivity as a possible mechanism behind sleep disruptions. At the core of qualitative research lies the gathering of in-depth material, providing rich and detailed testimonies about participants' experiences (Sandelowski, 1996). We chose to implement qualitative method in this study in order to obtain detailed insight into athletes' own perceptions of the studied variables, stress and sleep, which are currently not available in the literature on junior athletes. Furthermore, as the current qualitative study was part of a larger quantitative research project (Hrozanova et al., 2020), we supported the qualitative material with data from relevant questionnaires.

**Figure 1:**
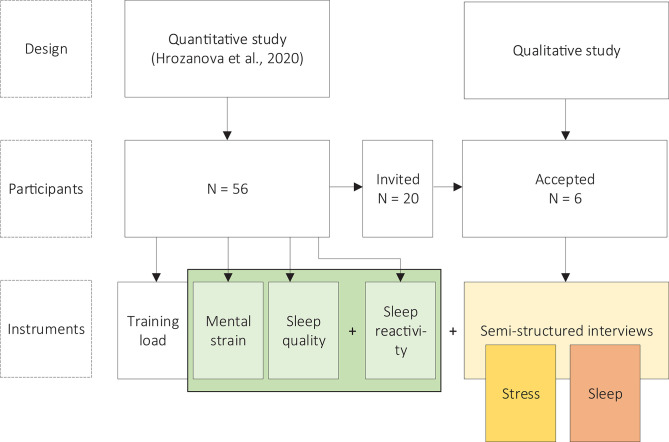
An overview of the design, participants, and variables investigated in the current study (yellow/orange boxes), and how these relate to the quantitative (green box) study by Hrozanova et al. (2020).

The quantitative study by Hrozanova et al. (2020) spanned 61 consecutive days, and included 56 junior endurance athletes from three high schools specialized for elite sports in Norway. In this study, we collected daily measures of objective sleep, training load and mental strain, in addition to a single measure of subjective sleep quality to identify good and poor sleepers in the sample. Although unreported in Hrozanova et al. (2020), the study also obtained a measure of sleep reactivity from all participating athletes. The current qualitative study took place at the end of the quantitative data collection. Semi-structured interviews were conducted to explore athletes' own perceptions of sleep and stress, and how these interact to impact athletic functioning. We included the quantitative measures of subjective sleep quality and mental strain from the Hrozanova et al. (2020) study, as well as the previously unreported measures of sleep reactivity, to supplement athletes' own accounts of stress and sleep quality. The overview of the design, procedure and investigated variables in the current study is presented in Figure 1.

### Participants

To facilitate the goals of qualitative research, sample sizes generally tend to be small. While power analysis may be used in quantitative research to ascertain the needed number of participants, no such tools exist for qualitative research. Rather, sample sizes in qualitative research are *purposive* and stand in contrast to random probability sampling employed in quantitative studies. Purposive sampling refers to the selection of participants based on their capacity and willingness to provide in depth, rich information on a given investigated topic (Luborsky and Rubinstein, 1995). It has previously been recommended that rather than following clear-cut guidelines on the number of participants in qualitative research, the focus should be on obtaining thorough, detailed and highly textured responses from each person (Morse, 2000). According to Sandelowski (1996), rich quantitative material should allow for new understanding to unfold, but it should be kept small enough that the detailed, deep analysis of the vast quantitative data is still possible.

Of the 56 junior endurance athletes from three high schools specialized for elite sports in Norway (Hrozanova et al., 2020), 20 athletes from one specific school were invited to participate in the current qualitative investigation. All 20 athletes received information about the purpose and aim of the qualitative investigation, and the voluntary nature of participation. Those who showed interest were included in the study. Of the 20 athletes, six volunteered to participate in this exploratory qualitative investigation. This gave the response rate of 30%. Three of the participating athletes were women and three men. Five athletes practiced cross-country skiing, while one practiced biathlon. Mean age was 17.7 ± 0.5 years.

### Ethics Statement

All athletes gave their informed consent to participate. Since all were above 16 years, parental consent was not necessary. The Regional Committee for Medical and Health Research Ethics in Central Norway approved the study (project ID 2017/2072/REK Central Norway).

### Instruments

#### Pittsburgh Sleep Quality Index (PSQI)

The PSQI (Buysse et al., 1989) measures retrospective sleep quality of the past month, calculated from 19 items focused on subjective recall of own sleep patterns, sleep disturbances, and the influence of sleep on daytime functioning. The questionnaire consists of seven components: **s**ubjective sleep quality, sleep latency, sleep duration, habitual sleep efficiency, sleep disturbances, use of sleeping medication, and daytime dysfunction. Of the 19 questions, four are scored from free entry answers, while the rest utilizes a 4-point Likert scale. The composite PSQI score ranges from 0 to 21, with values ≤5 indicating good sleep quality and values >5 poor sleep quality. The Norwegian version of the PSQI, with good psychometric properties, was used (Pallesen et al., 2005).

#### Ford Insomnia Response to Stress Test (FIRST)

The FIRST (Drake et al., 2004) is a nine-item questionnaire on stress-related propensity for sleep disturbance. The questions assess the degree of sleep difficulty a person typically experiences in response to nine potentially stressful scenarios, including before an important meeting the next day, after a stressful experience during the day and in the evening, after getting bad news, after watching a frightening movie, after a bad day at work, after an argument, before public speaking, and before going on vacation. Questions are scored on a 4-point Likert scale, ranging from 1 (not likely) to 4 (very likely). Responses are summated to generate the total FIRST score. An English version of the 9-item FIRST was used for back-to-back translations into Norwegian (Pallesen and Hrozanova, 2019). The Cronbach's alpha of the Norwegian version of the FIRST was 0.85.

#### Mental Strain

The assessment of mental strain was based on athletes' subjective reports of mood, and worry/rumination at bedtime. Athletes reported their mood and worry/rumination daily throughout the 61-days duration of the Hrozanova et al. (2020) study. For the reporting, we utilized an app-based visual analog scale (VAS) that ranged from 0 (no strain) to 10 (maximal strain). The final variable was calculated by averaging the mood and worry/rumination variable scores to create a measure of mental strain with both affective (mood) and cognitive (worry/rumination) components. This method for assessing mental strain was simple and time effective, which was crucial since athletes reported their scores every day for 61 consecutive days. For more details of the procedure, refer to Hrozanova et al. (2020).

#### Semi-structured Interviews

Six in-depth semi-structured interviews, ranging from 55 to 70 min, were conducted. Before the data collection, a three-stage guide for the interviews, based on the interviewers' theoretical knowledge and applied praxis in the field, was outlined. Firstly, we investigated athletes' own perception of sleep quality, quantity and sleep hygiene over the past 8 weeks. Secondly, questions investigating athletes' perceptions of own stress, relevant sources of stress and the ways athletes tackle stressful situations were asked. In this stage, the focus was quite broad, and elaborated on the themes the athletes themselves considered important and relevant, including the interactions between stress and sleep. Lastly, we explored other potentially influential factors on athletes' mental states and well-being (e.g., social relations, school, daily hassles). All questions were open-ended, and the interviews had a conversational tone. Question order was kept flexible in order to follow participants' perspectives, and researchers probed areas of interest to the problem statement through all stages.

### Data Analysis

The Grounded Theory by Charmaz (2006) was used to analyze the qualitative material. Interviews were audiotaped and transcribed to obtain an accurate basis for analysis. Researchers ensured thorough familiarity with the data. Coding, the pivotal link between collecting data and developing an emergent theory to explain the material, was initiated by identifying segments of text with meaningful utterances. Each utterance was marked, and its core idea considered. Then, one or more code words were attached to the relevant utterances, yielding conceptual components which were noted in a table to create an overview of the concepts found in the material. Material identified through coding was then interpreted in light of theoretical concepts related to sleep and stress using the constant-comparative methodology. This process involved theorizing about how each utterance related to the larger concepts investigated in the current study, forming theories about the material. Finally, categorization was utilized to gather information about the same theme within its respective category. During this stage, utterances identified through coding and theories identified in the constant-comparative process were grouped into main categories. Negative case analysis was utilized to remove the utterances that did not fit into the main theoretical categories. Each main identified category represented a specific theoretical model.

The quantitative material, comprising of PSQI, FRIST and mental strain scale, was descriptively summarized using IBM SPSS (version 27.0). The composite scores for PSQI and FIRST were reported. As mental strain was assessed continuously over a 61-days period, each participants' mental strain score was calculated as mean ± standard deviation (SD) of the entire 61-days period. The PSQI was used to distinguish between good and poor sleepers, by categorizing all individuals with scores >5 as poor sleepers (Buysse et al., 1989). In addition, median values of each participant's FIRST and mental strain scores were calculated to distinguish between participants scoring low and high on these measures. As these quantitative data only served a supplementary role in this study, and as the number of participants was low, no statistical analyses were conducted.

### Credibility and Trustworthiness

During the data collection, paraphrasing with phrases as “If I understand you correctly…” or “It sounds like…” gave participants the opportunity to elaborate on their experiences, delve more deeply into certain topics, or correct their message in case of misunderstandings (Coyle, 1998; Louw et al., 2011). Member checking represented another such opportunity. Each participant was e-mailed a copy of their transcribed interview and was encouraged to read through, and if desired, to change, amend or clarify its contents to ensure an accurate reflection of athletes' experiences and ideas (Smith and McGannon, 2018).

In order for participants' experiences and ideas to become evident, and to prevent the researchers from bringing own views or meanings into the data (Yardley, 2017), an independent researcher was hired to transcribe the interviews. Further, peer debriefing, or a discussion about the core meanings in the data and its final thematic structure among the researchers, was used to ensure the validity of the results (Jones et al., 2012). Researchers agreed on which utterances best exemplified the core meanings in the respective categories, evidencing a fit between the data and its allocated meaning. Thus, all researchers were involved in coding, result interpretation, category and sub-category identification and choice of utterances.

## Results

### Main Qualitative Results

**Figure 2:**
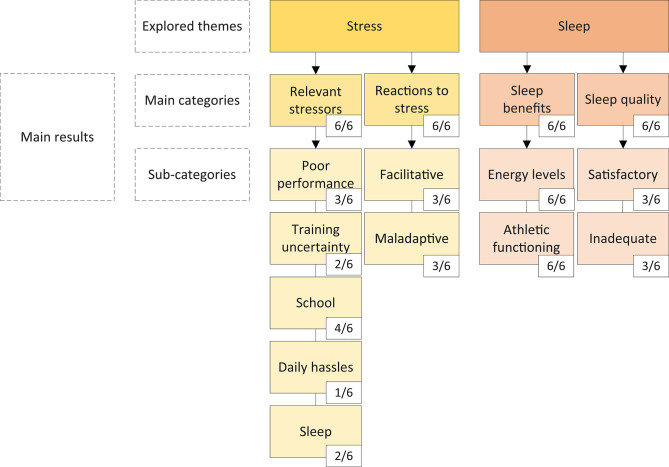
An overview of explored themes in the current qualitative study, the main categories, and sub-categories identified and their incidence in the semi-structured interviews.

Based on the semi-structured interviews that explored the themes of stress and sleep, and subsequent analysis based on the Grounded Theory (Charmaz, 2006), the following main results were identified. Two main categories, which all athletes reported experiencing, emerged in each explored theme. The main categories of the stress theme included “relevant stressors” and “reactions to stress,” and the main categories of the sleep theme included “sleep benefits” and “sleep quality.” Furthermore, multiple sub-categories emerged from each of the main categories. The frequency of the identified sub-categories varied among participants. “Poor performance,” “uncertainty in relation to training,” “school,” “daily hassles,” and “sleep” constituted the relevant stressors. Not all athletes experienced all identified relevant stressors, but each athlete reported experiencing at least two, with the stressors “poor performance” and “school” reported most commonly. The “reactions to stress” category included the sub-categories “facilitative” and “maladaptive.” Half the sample experienced facilitative, while the other half experienced maladaptive reactions to stress. “Energy levels” and “athletic functioning” constituted the “sleep benefits” category, and “sleep quality” included the sub-categories “satisfactory” and “inadequate.” All athletes perceived that sleep improved their energy levels and athletic functioning, while half the sample experienced satisfactory sleep quality, and the other half experienced inadequate sleep quality. The main results, with incidence of the identified categories and sub-categories, are shown in Figure 2. Relevant utterances supporting the division into sub-categories of the stress and sleep themes are presented in Tables 1, 2, respectively.

**Table 1:** The identified stress categories and sub-categories based on semi-structured interviews with six junior endurance athletes, with representative utterances.

**Main categories**	**Sub-categories**	**Utterances**
Relevant stressors	Poor performance	“…It makes me quickly a bit stressed when training or results in training sessions or competitions are not as I wished for. I have a big focus on cross-country skiing and it means everything to me.”–P6 “I feel that I am standing still, whatever I try and however hard I'm training, or however much I'm training, there's not much effect. I'm working on it but it's a bit like running in water in a way. There's no traction, there's no progress forward.”–P5
	Uncertainty in relation to training	“…I got told by my coach to try to start training again [after a training hiatus]. To loosen it [the body] up a bit. But it never did, and I was very unsure. My body just told me to rest to get surplus of energy again. I tried to rest but my coach told me that was wrong, the coach wanted one thing and I wanted the other. It was difficult for me to know what to do.”–P6 “That [what I could do differently in relation to poor performance] I have thought of many times. I have tried to figure it out. I had two downward spirals before. I can't figure out what to take away or do differently.”–P5
	School	“…I can feel that it's difficult to take the books out after I come home from training, and start with homework. If I have to do it [homework], then I typically do it before training. If I'm training then I have to go to bed at the right time, so I cannot sit and fiddle with homework until late at night.”–P4 “I'd like for everything to be perfect. Or actually, the things I do at school and biathlon. Everything else doesn't have to be so perfect. But I'm quite thorough with those two things. I'm very worried if I'm feeling like I have not studied enough for an exam. This makes me stressed a lot.”–P2
	Daily hassles	“…It's never like a week can go by with me feeling that everything is on track. It never is.”–P5
	Sleep	“…I get stressed when I am awake. Then it also takes longer to fall back to sleep. I look at the clock and I think, well, now I should have been sleeping. And that makes me stressed.”–P2 “It is very much that I know that if I don't get enough sleep, then the next day will be a little ruined. I know that that will probably contribute to training going badly, and since I care so much about biathlon… I think it ends up with that I am scared that sleep will influence biathlon.”–P2
Reactions to stress	Facilitative	“…I know what I'm good at. I know that I am not actually 4 min behind the guy that won. So it's alright when I feel in my body that I can't [go on] anymore. Then I know that I'm having a difficult day, and I just focus on a new opportunity.”–P3 “…I am not typically stressed when I compete in a ski race. We typically have some focus areas on races, so that I don't think about the places on the podium. I only think about the technical stuff, or the goals I have set for myself, there's no time for the rest.”–P1 “… I just had to keep calm when I got sick. I think that if you start with worrying about getting sick then you will be stressed by it too. You will get sick from thinking that you don't want to get sick.”–P4
	Maladaptive	“…I probably think a bit too much about biathlon. It's not often I *don't* think about it… If it goes badly at a ski race then I'm thinking–everybody has seen that. … I am probably a bit worried about what others think of things I do when it comes to biathlon.”–P2 “…I have always in a way been a strong person, a person who has managed this. I can manage this–[…] making food, […] waxing skis, […] doing this by myself. But I have now in a way maybe understood a bit that I'm having a difficult time. … I feel like it's the opportunity to calm down that I don't have. … To be in balance, I don't even remember what that is anymore.”–P5 … I think a lot about everything to do with performing. I have a wish to go fast on skis. So when it doesn't happen, I can get a bit caught up in it. What can the reason be and bla, bla, bla. I think about these things when I'm falling asleep.”–P6

**Table 2:** The identified sleep perception categories and sub-categories based on semi-structured interviews with six junior endurance athletes, with representative utterances.

**Main categories**	**Sub-categories**	**Utterances**
Sleep benefits	Energy levels	“…I prioritize going to bed early so that I get as much energy as possible during the day.”–P1 “Focus and concentration are best when I sleep well. For me, sleep is as important as nutrition.”–P3
	Athletic functioning	“…I know the next day will be ruined if I don't get enough sleep. I know it adds to that maybe training goes badly, and since biathlon means the most to me…”–P2 “…I have always been dependent on good sleep. In the periods when I sleep poorly, it impacts everything.”–P3
Sleep quality	Satisfactory	“…I sleep well. I am not awake much, fall asleep swiftly, maybe in about 10 min after I've put my phone away. In the morning, I get up straight away. I typically switch off the alarm and I get up and get dressed at once. So in 2 min I am completely awake.”–P3 “…I myself think that I sleep well. I don't have any problems with sleep, to put it this way. I am functioning well during the daytime and I'm in continuously good shape during the day.”–P4
	Inadequate	“…I get tired, I want to sleep at around 8 PM and when it gets close to 10 p.m. I feel completely awake. …I'm lying in bed, awake with my phone, in long stretches of time. I have a bad habit of using my phone before I go to bed. When I put it [the phone] away I cannot sleep, it can typically take between half an hour to an hour to fall asleep.”–P6 “…I can wake up 4–5 times during the night. I don't feel like I sleep particularly deeply, it doesn't take much sound for me to wake up. …and it generally takes quite a long time before I fall back to sleep again.”–P2

### Supplementary Descriptive and Quantitative Data

The descriptive statistics of each participant's sex, age, practiced sport, composite PSQI score, FIRST and mental strain score are presented in Table 3. Three of the participants in this study (P1, P3, P4) scored ≤5 on the PSQI (Buysse et al., 1989), and were thus categorized as good sleepers. The remaining participants (P2, P5, P6) were categorized as poor sleepers, due to their PSQI scores being above the cutoff of five (Buysse et al., 1989). The median value for FIRST was 12.5 and 3.1 for mental strain. Therefore, participants P1, P3, and P4 were in the lower scoring group on both FIRST and mental strain, while participants P2, P5, and P6 were in the higher scoring group.

**Table 3:** The sex, age, practiced sport, composite PSQI score, FIRST score and mental strain values of each participant.

**Participant**	**Sex**	**Age**	**Sport**	**PSQI**	**FIRST**	**Mental strain**
P1	Woman	17	Cross-country skiing	2	12	2.5 ± 0.3
P2	Woman	18	Biathlon	9	23	3.7 ± 0.2
P3	Man	18	Cross-country skiing	2	9	2.1 ± 0.2
P4	Man	18	Cross-country skiing	3	10	2.0 ± 0.1
P5	Woman	18	Cross-country skiing	6	13	5.2 ± 0.2
P6	Man	17	Cross-country skiing	9	29	3.7 ± 0.3

*PSQI, Pittsburgh Sleep Quality Index; FIRST, Ford Insomnia Response to Stress Test*.

### Associations Between Stress and Sleep

**Figure 3:**
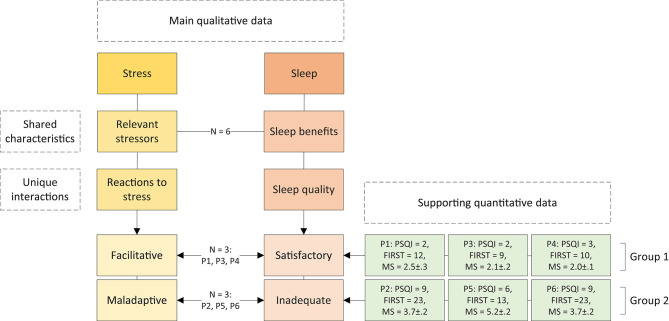
Breakdown of the two groups that emerged when identifying associations between stress and sleep: Group 1 with facilitative reactions to stress and satisfactory sleep quality, and Group 2 with maladaptive reaction to stress and inadequate sleep quality. Pittsburgh Sleep Quality Index (PSQI), Ford Insomnia Response to Stress Test (FIRST), and mental strain (MS) values of each participant are reported.

Two distinct associations between stress and sleep emerged in the sample. Three of the interviewed athletes who experienced facilitative reactions to stress exhibited satisfactory sleep quality (Group 1: P1, P3, P4), and the three athletes who experienced maladaptive reactions to stress exhibited inadequate sleep quality (Group 2: P2, P5, P6). These main results, identified from the semi-structured interviews, may be supplemented by the available quantitative data. All participants belonging to Group 1 were considered good sleepers based on the PSQI. In addition, these participants scored in the lower range of both FIRST and mental strain scales. On the other hand, all participants belonging to Group 2 were considered poor sleepers based on the PSQI, and scored in the higher range of both FIRST and mental strain scales. See Figure 3 for a breakdown of the two groups, including participant number, category of reaction to stress and sleep quality, and values on the PSQI, FIRST, and mental strain scales.

## Discussion

The present study performed semi-structured interviews in six junior endurance athletes to qualitatively investigate their perceptions of stress and sleep, and the associations between these variables. The first main finding showed that stress could be differentiated into “relevant stressors” (encompassing “poor performance,” “uncertainty in relation to training,” “school,” “daily hassles,” and “sleep”) and “reactions to stress” (with sub-categories “facilitative” and “maladaptive”). The second main finding showed that sleep could be differentiated into “sleep benefits” (encompassing “energy levels” and “athletic functioning”) and “sleep quality” (with sub-categories “satisfactory” and “inadequate”). While all athletes identified relevant stressors and all understood the importance of sleep for athletic functioning, these results also point to differences between athletes based on their reactions to stress and sleep quality: one group of athletes exhibited facilitative reactions to stress and satisfactory sleep quality, while the other exhibited maladaptive reactions to stress and inadequate sleep quality. These findings were supplemented by quantitative data on subjective sleep quality based on the PSQI, sleep reactivity based on the FIRST, and mental strain based on a VAS scale. The first group of athletes, who experienced facilitative reactions to stress and satisfactory sleep quality, scored as good sleepers on the PSQI, and in the lower ranges of the FIRST and VAS. The second group of athletes, who experienced maladaptive facilitative reactions to stress and inadequate sleep quality, scored as poor sleepers on the PSQI, and in the upper ranges of the FIRST and VAS.

### Stress in the Athletic Setting

For junior athletes, the exposure to stress is an inevitable part of daily functioning. Therefore, the initial focal point of the interviews was not whether athletes experienced stress, but rather what caused the stress that occurred. Relevant stressors included “poor performance,” “uncertainty in relation to training,” “school,” “daily hassles” and “sleep.” Each athlete reported experiencing at least two of the reported stressor categories, suggesting that athletes experience many of the same stressors. Such stressors may arise frequently (Brantley et al., 1987), and may heavily influence a person's well-being (DeLongis et al., 1982), reflecting the stressors' strong impact on some of the individuals in this study.

The stressors identified in the current study fit closely with the three major stressor categories established by Nixdorf et al. (2015): Double burden, sport-specific demands and conditions. Stressors “school” and “daily hassles” fit into the double burden category of combining sports with school and other activities, which could be exemplified with the utterance from Participant 4: “…I can feel that it's difficult to take the books out after I come home from training, and start with homework. If I have to do it [homework], then I typically do it before training. If I'm training then I have to go to bed at the right time, so I cannot sit and fiddle with homework until late at night..” The stressors “sleep” and “poor performance” were reported in relation to the demands of the athletes' sport. For instance, Participant 2 said: “… I know that if I don't get enough sleep, then the next day will be a little ruined. I know that that will probably contribute to training going badly, and since I care so much about biathlon… I think it ends up with that I am scared that sleep will influence biathlon..” This illustrates that sleep was viewed as a stressor that might influence the participants' sport performances, and we therefore postulate that sleep may be conceptualized as a stressor in the sport-specific demands category. Lastly, the stressor “uncertainty in relation to training” fit in Nixdorf et al.' category conditions, as athletes in this study related this stressor to unfavorable structures within the team or organization, exemplified here by a conflict with a coach: “…I got told by my coach to try to start training again [after a training hiatus]. To loosen it [the body] up a bit. But it never did, and I was very unsure. My body just told me to rest to get surplus of energy again. I tried to rest but my coach told me that was wrong, the coach wanted one thing and I wanted the other. It was difficult for me to know what to do (Participant 6).”

Having established the relevant stressors, the interviews focused on athletes” reactions to stress. Athletes reacted to stressors in two distinct ways—facilitatively and maladaptively. Three athletes kept a rational, positive mindset and stayed in control of the situation when exposed to a relevant stressor. According to the BPS, these athletes exhibited a facilitative reaction to stress as they perceived their coping resources to be adequate for the management of the stressful event (Blascovich et al., 2001). Such facilitative reaction to stress may be illustrated by the utterance from Participant 3: “…I know what I'm good at. I know that I am not actually 4 min behind the guy that won. So it's alright when I feel in my body that I can't [go on] anymore. Then I know that I'm having a difficult day, and I just focus on a new opportunity.” The three other athletes reacted to stressors with worry, rumination, sense of overwhelm and overthinking, as well as seeking external validation. In terms of the BPS, they possessed inadequate coping resources to manage the stressful situation, and thus exhibited a maladaptive reaction to stress (Blascovich, 2008). Such reaction may be illustrated by the utterance from Participant 5: “…I have always in a way been a strong person, a person who has managed this. I can manage this—[…] making food, […] waxing skis, […] doing this by myself. But I have now in a way maybe understood a bit that I'm having a difficult time. … I feel like it's the opportunity to calm down that I don't have. … To be in balance, I don't even remember what that is anymore.”

Important to the interpretation of these findings may be the metacognitive theory of detached mindfulness (Wells, 2005), which considers athletes' awareness of their own thoughts and beliefs about the stressful situations and their attempts to react by acceptance or suppression. According to the detached mindfulness framework, athletes exhibited facilitative reactions to stress likely due to their ability to detach their attention from potential stressors and to avoid dwelling on dysfunctional thoughts and preservative cognitions (worries and ruminations), in relation to the stressor. Evidence comes, among others, from Participant 4: “… I just had to keep calm when I got sick. I think that if you start with worrying about getting sick then you will be stressed by it too. You will get sick from thinking that you don't want to get sick ….” Therefore, for athletes who responded to stressors facilitatively, detached mindfulness seems to be a conscious act that results from their awareness of own thinking. This allows athletes to perceive the event as merely a situation that will pass. On the other hand, athletes who exhibited maladaptive reactions to stress were seemingly unable to detach their attention from the stressors. Rather, they fixated their attention on the relevant stressor, which lead to worrying and rumination. They also demonstrated heightened focus on threat monitoring, which is central to the experience of maladaptive reactions to stress. This may be illustrated by utterance from Participant 6: “… I think a lot about everything to do with performing. I have a wish to go fast on skis. So when it doesn't happen, I can get a bit caught up in it.” Athletes in this group seemed to focus considerable attention and cognitive processing on attempting to manage and solve the stressors they faced. As the results show, athletes' abilities to consciously decide on how they react to a stressful situation may be crucial in giving (dys-)functional thoughts their salience, and to determine the outcome of the stress response (Wells, 2005).

### Sleep in Athletes, and Associations to Stress

Addressing sleep, the two main identified categories were “sleep benefits” and “sleep quality.” The “sleep benefits” category revealed that all athletes are aware of the importance of adequate sleep for energy levels and athletic functioning, which may be illustrated by the following utterance from Participant 3: “…I prioritize going to bed early so that I get as much energy as possible during the day.” However, such awareness alone does not guarantee that athletes sleep well. Indeed, the semi-structured interviews uncovered that half of the sample experienced “inadequate” sleep quality, while the other half perceived “satisfactory” sleep quality. This was largely due to nightly awakenings, intrusive thoughts at bedtime, difficulty initiating sleep, short sleep durations, poor sleep hygiene and tiredness upon awakening. This may be illustrated by the utterance from Participant 6 (and Participant 2, see Table 2): “…I get tired, I want to sleep at around 8 p.m. and when it gets close to 10 p.m. I feel completely awake. …I'm lying in bed, awake with my phone, in long stretches of time. I have a bad habit of using my phone before I go to bed. When I put it [the phone] away I cannot sleep, it can typically take between half an hour to an hour to fall asleep.” Such subjective sleep disruptions are known to occur in athletic populations (Bender et al., 2018). The experience of poor sleep quality was underpinned by the results of the PSQI: The participants who experienced inadequate sleep quality could be categorized as poor sleepers based on the index, while those who experienced satisfactory sleep quality could be categorized as good sleepers.

Strikingly, the three athletes that experienced satisfactory sleep quality exhibited facilitative reactions to stress (Group 1), while the other three athletes experienced inadequate sleep quality and maladaptive reactions to stress (Group 2). Links between stress and sleep, in either or both directions, exist in various populations (Hall et al., 2000; Harvey, 2000; Morin et al., 2003; Kahn et al., 2013; Hrozanova et al., 2020). The concept of sleep reactivity has been highlighted as a fundamental mechanism describing the propensity of individuals to stress-related sleep disruptions (Drake et al., 2014). The supplementary quantitative data showed that the athletes belonging to Group 1 exhibited low sleep reactivity, while the athletes belonging to Group 2 exhibited high sleep reactivity. Likewise, the mental strain scale, which measured the cognitive and affective components of the stress response (i.e., mood and preservative cognitions worry/rumination at bedtime) showed that athletes from Group 1 scored low on mental strain, while athletes from Group 2 scored high on mental strain. Thus, the overall pattern that emerged from these data indicates that athletes who perceive their own sleep quality to be satisfactory, and who experience facilitative reactions to stress, are objectively classified as good sleepers (PSQI), have low propensity toward sleep reactivity (FIRST), and low levels of mental strain (VAS). On the other hand, athletes who perceive their own sleep quality to be inadequate, and who experience maladaptive reactions to stress, are objectively classified as poor sleepers (PSQI), have higher propensity toward sleep reactivity (FIRST), and higher levels of mental strain (VAS). A link between reactions to stress and sleep quality thus emerges, with important roles played by sleep reactivity and preservative cognitions.

Sleep reactivity and preservative cognitions that the poor sleepers in this study engaged in likely play important roles in the interaction between stress and sleep. In the current study, athletes who engaged in preservative cognitions at bedtime (mental strain) had higher propensity toward sleep reactivity, and experienced maladaptive reactions to stress as well as poor sleep quality. These associations emerged even though the exposure to stressors was comparable across all athletes. Previously, individuals with high sleep reactivity that engage in preservative cognitions were found to be especially prone to sleep disturbances (Drake et al., 2014). Based on our findings and previous research, we propose that both preservative cognitions and sleep reactivity represent maintaining mechanisms of the stress response, prolonging it and ultimately feeding into the development of poor sleep. In junior athletes, worry inversely predicts sleep quality (Hrozanova et al., 2019), and uniquely in poor sleepers, increases in mental strain at bedtime are associated with increased sleep onset latency (Hrozanova et al., 2020). In this context, preservative cognitions likely amplify the impact of the daily stresses, thereby causing sleep disruptions (Harvey, 2000). Indeed, in a study of good and poor sleepers who experienced comparable stress, the perceived impact of the stressors was greater for poor than good sleepers. Poor sleepers engaged in preservative cognitions, viewed their daily lives as more unpredictable, and with more uncontrollable situations than the good sleepers (Morin et al., 2003). These findings implicate preservative cognitions, which occur in maladaptive reactions to stress, in the experience of poor sleep quality.

The stress-sleep interactions in this study indicate that some athletes may be at risk of developing a self-reinforcing cycle of poor sleep and maladaptive reactions to stress. In the present study, athletes understood that optimal sleep was important for their functioning. In some individuals, a persistent focus on good sleep may become a stressor, which is ultimately detrimental to achieving good sleep (Baron et al., 2017). The aforementioned utterance from Participant 3, who stated: “…I prioritize going to bed early so that I get as much energy as possible during the day,” may be on one hand seen as reflective of understanding that sleep is important for optimal functioning. However, it may also be a strategy to ensure adequate sleep. If this individual engages in preservative cognitions, and exhibits propensity toward sleep reactivity, such strategies may become maladaptive, as the desire for good quality sleep may become a stressor in itself. The identification of sleep as a sport-specific stressor was also seen in the current study. In addition, athletes reported worrying and ruminating about poor sleep and some also experienced poor sleep quality. These associations may increase athletes' need for recovery and sleep, which may be difficult to obtain in light of the preservative cognitions that maintain and prolong the stress response, preventing sleep of good quality. Ultimately, athletes may land in a negative cycle where both poor sleep and maladaptive reactions to stress reinforce one another.

### Practical Implications and Future Directions

The current study reveals novel insight into the associations between sleep and stress in a sample of junior athletes, and suggests that reactions to stress, the tendency toward sleep reactivity and engagement in preservative cognitions may play important role**s** in the sleep disturbances experienced by this sample. However, the current results are drawn from a small sample. Future research should investigate the relevant constructs with quantitative measures, in a larger sample from which conclusions about junior athletes may be drawn. Importantly, it may be important to utilize ecological assessments of sleep and stress to obtain accurate quantitative insights. Both preparatory and competitive phases of the season should be investigated, as each of these phases place unique demands on the junior athletes.

In the current study, athletes' facilitative reactions to stress occurred alongside good sleep quality, based on both subjective perception and the PSQI; as well as low propensity toward sleep reactivity and low cognitive and affective mental strain at bedtime. These findings may have important practical implications. Having the ability to evaluate stressful events as challenging instead of threatening, seeing the stressors as opportunities for self-growth, and the ability to practice detached mindfulness, may be crucial for withstanding the maladaptive outcomes of the stress response. Previously, facilitative reactions to stress have been associated with high mental resilience (Fletcher and Sarkar, 2012). When compared to insomniacs, good sleepers had higher mental resilience, and lower sleep reactivity and hyperarousal (Palagini et al., 2018). Moreover, high resilience was quoted as a possible reason for the low sleep reactivity among elite athletes in the study by Gupta et al. (2017). Similarly, mental resilience was shown to predict better sleep, based on both objective (non-athletes, mean age 18.9 years, in Brand et al., 2014) and subjective (junior athletes, mean age 18 years, in Hrozanova et al., 2019) parameters. In the latter study of junior athletes, two mental resilience factors, social resources and structured style [i.e., an individual's tendency to approach daily routines and goals in a planned, structured manner (Friborg et al., 2005)], were protective of sleep quality (Hrozanova et al., 2019). Thus, an important practical implication of the current research could involve making better use or improving athletes' mental resilience.

In a stressful situation, an important source of social resources and structured style may be a supportive coach. Supportive coaches can influence junior athletes' well-being by eliminating achievement-related preservative cognitions (Ommundsen et al., 2006). In one study, this was found to be helpful when coping with sport-related stressors (Kristiansen and Roberts, 2010). Adequate social resources from the coach may aid the athlete in increasing coping resources, and decreasing perceived situational demands, helping the athlete to see the positive opportunities presented by a potentially threatening situation. Such shift in perspective may be helpful in perceiving a stressor as a challenge in place of threat, which may ultimately have consequences for not only the stress response, but also sleep reactivity and sleep quality. Therefore, future research should focus on understanding the coach-athlete relationship, exploring what it takes for a coach to be perceived as supportive, and investigate whether athletes' mental resilience may be utilized to a greater extent in a relationship with a supportive coach.

Future research should also investigate ways of improving athletes' own mental resilience independent of the coach. Such interventions may be especially useful for junior athletes, as juniors have lower ability to cope with adversity than senior athletes (Bebetsos and Antoniou, 2003). Relevant interventions may be built on the cognitive-behavioral approach, utilizing, for instance, aspects of the metacognitive theory of detached mindfulness, in order to increase awareness of own thoughts and beliefs about stressful situations, and to encourage attempts to react to stressors by acceptance rather than suppression (Wells, 2005). Previous research has shown that group, school-based programs developed to increase mental resilience in adolescents lead to reduction in depressive symptoms (Brunwasser et al., 2009). In college athletes, an expressive-writing course to increase mental resilience lead to improved decision making, decreased perceived stress, and higher resilience when compared to controls (Chandler et al., 2020). Mental resilience-enhancing interventions should be in the future developed specifically for junior athletes, to target the stressors and challenges typical for this population.

## Limitations

The present results should be interpreted with some limitations in mind. Firstly, the sample size included only six participants. Although this is deemed adequate in qualitative research (Dukes, 1984), the small sample size prevents us from drawing any definite conclusions about the associations discussed in this study. Moreover, the small sample size prevents us from analyzing the supplementary quantitative data statistically, due to concerns of low power and inadequate representation of the sample. Secondly, the nature of the qualitative data is retrospective, and it does not give the possibility to establish any causal relationships between sleep quality and reactions to stress. In addition, some participants may have left out particularly sensitive, emotional or distressing information from their narratives. It is also important to point out that in the semi-structured interviews, we did not specifically ask about hyperarousal, or mental resilience. Athletes may not have regarded these aspects as important when talking about sleep and stress, and therefore, they did not emerge clearly from the qualitative material. Moreover, athletes were not screened for clinical sleep disorders, which might have been relevant for the three athletes experiencing poor sleep quality. Finally, utterances were translated from Norwegian to English. Despite our best efforts not to amend the meanings, it should be kept in mind that these are in fact translations and not original utterances.

## Conclusion

Based on in-dept qualitative material and supplementary quantitative data, the present findings provide novel indications of the potential interactions between stress and sleep in junior endurance athletes. Keeping in mind the limited sample size, we have found that all athletes identified relevant stressors, and all were aware of the importance of sleep for their athletic functioning. However, athletes formed two distinctive groups based on the interactions between stress and sleep: one group exhibited facilitative reactions to stress and good sleep quality, as well as low propensity to sleep reactivity and low mental strain. Meanwhile, the other group exhibited maladaptive reactions to stress and poor sleep quality, as well as high propensity to sleep reactivity and high mental strain. It seems that it is not simply the isolated stress exposure, but rather, athletes' predisposition to sleep disturbance, the way the relevant stressors are evaluated, and the salience that is given to the (dys-)functional thoughts, that plays a major role in the relationship between stress and sleep. Keeping the reciprocal associations between sleep and stress in mind, it is likely that poor sleep quality and maladaptive stress responses may form a negative cycle, which may exacerbate sleep difficulties. Conceptualizing sleep quality based on the evaluation of stressors may thus offer a plausible explanation for why the occurrence of stressors leads to poor sleep quality in some individuals, but not others. Future research should further investigate the concepts of reactions to stress, sleep reactivity, and sleep quality on a larger scale, ideally utilizing ecological assessment of the constructs over longer time periods.

## Data Availability Statement

The raw data supporting the conclusions of this article will be made available by the authors, without undue reservation.

## Ethics Statement

The studies involving human participants were reviewed and approved by The Regional Committee for Medical and Health Research Ethics in Central Norway. Written informed consent from the participants' legal guardian/next of kin was not required to participate in this study in accordance with the national legislation and the institutional requirements.

## Author Contributions

MH and FM designed, conceptualized the study, and collected the data. FM facilitated contact with the athletes involved in this study. KF, MH, and FM engaged in data analysis and interpretation of data. MH wrote the manuscript. KF and FM contributed to the writing and editing of the manuscript. The final version of the manuscript was approved by all authors.

## Conflict of Interest

The authors declare that the research was conducted in the absence of any commercial or financial relationships that could be construed as a potential conflict of interest.
